# A 15-year-old adolescent with a rare pituitary lesion

**DOI:** 10.1530/EDM-14-0010

**Published:** 2014-05-01

**Authors:** Despoina Manousaki, Cheri Deal, Jean Jacques De Bruycker, Philippe Ovetchkine, Claude Mercier, Nathalie Alos

**Affiliations:** 1 Department of Pediatrics, Endocrine Service, Centre Hospitalier Universitaire Sainte Justine and Université de MontréalMontreal, QuebecCanada; 2 Department of Pediatrics, Immunology Service, Centre Hospitalier Universitaire Sainte Justine and Université de MontréalMontreal, QuebecCanada; 3 Infectious Disease Division, Department of Pediatrics, Centre Hospitalier Universitaire Sainte Justine and Université de MontréalMontreal, QuebecCanada; 4 Surgery Department and Chief of Neurosurgery Service, Centre Hospitalier Universitaire Sainte Justine and Université de MontréalMontreal, QuebecCanada

## Abstract

Cystic sellar lesions are a rare cause of hypopituitarism and extremely rare in the pediatric age group. The differential diagnosis is large and includes both primary pituitary abscesses and cystic components on pre-existing lesions, such as adenoma, craniopharyngioma, Rathke's cleft cyst, leukemia, granulomatous disease and lymphocytic hypophysitis. In the absence of a definitive diagnosis, treatment can be challenging. We report a case of a 15-year-old female, who presented with headaches, altered consciousness and diplopia after a molar extraction, for which she had received oral antibiotics. Broad-spectrum i.v. antibiotics were given for presumed meningitis. Blood cultures failed to identify pathogens. Cerebral magnetic resonance imaging showed a pituitary cystic lesion. Endocrine studies revealed abnormal pituitary function. In the absence of a therapeutic response, the patient underwent a transsphenoidal biopsy of the pituitary gland, which yielded a purulent liquid, but cultures were negative. Histopathology showed lymphocytic infiltrates but no neutrophils, compatible with an inflammation of autoimmune or infectious origin. High-dose glucocorticoid therapy was started and pursued, along with i.v. antibiotics, for 6 weeks, leading to clinical and radiological improvement but with persistence of endocrine deficits. In conclusion, this is a case of secondary panhypopituitarism due to a cystic pituitary lesion, with a differential diagnosis of lymphocytic hypophysitis vs abscess in a context of decapitated meningitis. Combination therapy with antibiotics and glucocorticoids is a legitimate approach in the face of diagnostic uncertainty, given the morbidity, and even mortality, associated with these lesions.

**Learning points:**

- It is not always easy to differentiate primary cystic sellar lesions (such as a primary infectious pituitary abscess) from cystic components on pre-existing lesions (such as adenoma, craniopharyngioma, Rathke's cleft cyst, leukemia or lymphocytic hypophysitis).
- Because of the absence of specific symptoms and of immunohistochemical and serum markers, response to glucocorticoids can be the only way to differentiate lymphocytic hypophysitis from pituitary lesions of another origin. In addition, microbiological cultures are negative in 50% of cases of primary infectious sellar abscesses, thus the response to antibiotic treatment is often the key element to this diagnosis.
- A short course of high-dose glucocorticoids combined with antibiotics is not harmful in cases where there is no diagnostic certainty as to the origin of a cystic sellar mass, given the morbidity and mortality associated with these lesions.
- This approach may also diminish inflammation of either infectious or autoimmune origin while ensuring that the most likely pathogens are being targeted.

## Background

Cystic lesions represent 6% of all sellar lesions. Sellar abscesses of infectious origin are a rare cause of hypopituitarism. They can result from a pituitary-limited infection (primary sellar abscesses), through hematogenous extension (septicemia) or as a complication of meningitis. Secondary abscesses develop on pre-existing lesions (such as adenoma, craniopharyngioma, Rathke's cleft cyst, leukemia or lymphocytic hypophysitis) (1). Autoimmune or lymphocytic hypophysitis is an autoimmune phenomenon occurring primarily in women in the *postpartum* state. Cerebral imaging studies of cases of lymphocytic hypophysitis reveal a pear-shaped pituitary, with or without cystic components. Because of the absence of specific symptoms, immunohistochemical and serum markers and because of overlapping radiological findings, the differentiation of cystic sellar lesions can be difficult and the choice of therapeutic approach problematic. Often response to treatment is a key element to diagnosis. This case supports the use of combination therapy when confronted with this diagnostic dilemma.

## Case presentation

A previously healthy 15-year-old (Tanner 5) white female consulted for an altered state of consciousness and neck pain with a 2-week history of hemi-cranial headaches. Past medical history was significant for migraines. She underwent a molar extraction 6 days prior to her admission and received oral cloxacillin with transient relief followed by relapse of her symptoms. On admission, she was not febrile and her vital signs were normal, but due to a Glasgow score of 12, she was intubated and given broad-spectrum i.v. antibiotics (ceftriaxone and metronidazole). After 24 h, following extubation, she presented with horizontal diplopia and papillary edema.

## Investigation

The initial blood cell count was normal (leucocytes, 9.92×10^9^/l; platelets, 262×10^9^/l and hemoglobin 145 g/l) and erythrocyte sedimentation rate levels were mildly elevated (36 mm/h). The lumbar puncture showed the presence of white blood cells (leucocytes, 322/mm^3^; neutrophils, 10%; lymphocytes, 70% and erythrocytes, 7/mm^3^) and protein (1.17 g/l). No infectious agent was identified: Cerebro-spinal fluid (CSF) cultures remained negative for bacteria and fungi; specific cultures for mycobacterial infections were also negative.

The initial cerebral computed tomography with contrast was negative, apart from discontinuity in the cortex of the right posterior maxillary bone, secondary to the molar extraction.

**Figure 1:**
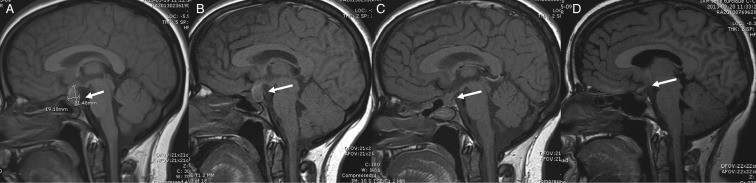
Cerebral MRI scans, sagittal T1. Arrows indicate the location of the mass. On admission and 1 week after, both pre-surgery (A and B): 1.7×1.1×1.9 cm rounded oval-shaped sellar lesion with suprasellar extension; immediately post-surgery (C) and 6 months post-surgery (D). We note the absence of the bright spot of the posterior pituitary on all four MRI scans. This can be predictive of a permanent central diabetes insipidus although specificity decreases with age.

A cerebral magnetic resonance imaging (MRI) scan revealed a 1.7×1.1×1.9 cm sellar lesion with suprasellar extension toward the floor of the third ventricle (Fig. 1A). The radiological diagnosis was pituitary apoplexy vs pituitary hemorrhage. One week later, a second cerebral MRI scan showed a thinning of the dorsum sellae (Fig. 1B) and a rounded oval-shaped lesion, isointense on T1-weighted images and iso-hyperintense on T2-weighted images, with a peripheral rim (Fig. 1B). This finding was compatible with a primary sellar abscess vs a secondary abscess on a pre-existing lesion (differential diagnosis: granuloma, craniopharyngioma, Rathke's cleft cyst and lymphocytic hypophysitis). In order to eliminate vasculitis as part of the investigation of an autoimmune origin of the lesion, a brain Fludeoxyglucose Positron Emission Tomography (FDG–PET) scan was performed and was negative. Anterior pituitary evaluation revealed impaired thyroid-stimulating hormone secretion with a low free thyroxine (free T_4_) and a low cortisol, consistent with hypopituitarism. A mildly elevated prolactin was consistent with pituitary stalk compression (Table 1). A complete immunological work-up (anti-nuclear, anti-neutrophil cytoplasmic, anti-smooth muscle, anti-gastric parietal cells, anti-mitochondrial, anti-DNA and anti-histone antibodies), as well as thyroid auto-antibodies were negative. IgG4 serum levels were normal. In the absence of diagnostic certitude and clinical improvement, pituitary biopsy was deemed essential.

**Table 1:** Results of baseline endocrine testing (reference ranges in parenthesis). For LH and FSH, reference ranges correspond to the follicular phase

****	
TSH (mIU/l)	0.04 (0.40–4.40)
Free T_4_ (μg/dl)	0.57 (0.69–1.05)
Cortisol (μg/dl)	0.46 (7–25)
LH (IU/l)	0.51 (6–30)
FSH (IU/l)	1.75 (4–20)
Estradiol (pg/ml)	1.38 (6–55)
DHEAS (μg/dl)	0.02 (0.05–0.32)
PRL (μg/l)	12.4 (0.0–11.1)
IGF1 (ng/ml)	221 (223–901)

The patient was started on replacement doses of oral hydrocortisone and l-T_4_ before her transsphenoidal aspiration biopsy, which took place 12 days after admission and yielded a purulent, cream-colored liquid. Microbiological cultures of the liquid remained negative. Histopathology of the pituitary gland biopsy showed diffuse lymphohistiocytic infiltrates, without granulomatous or giant cells, suggestive of an autoimmune or inflammatory process. The post-drainage MRI showed a net decrease in the pituitary lesion and a thickening of the pituitary stalk (Fig. 1C). The final diagnosis was pituitary abscess of an infectious vs an autoimmune origin.

## Treatment

I.v. methylprednisolone (1 g daily for 3 days), then high-dose oral glucocorticoids (dexamethasone for 3 days, then prednisone), was initiated upon reception of the results of the biopsy. Immediately after surgery, the patient developed persistent hypernatremia and polyuria compatible with diabetes insipidus and was started on oral desmopressin acetate. With improvement of her neurological status, she was discharged on oral desmopressin (desmopressin oral disintegrating tablet, 120 μg twice daily), l-T_4_ (75 μg daily), prednisone (50 mg daily) and i.v. meropenem. The antibiotic and prednisone were discontinued after 6 weeks.

## Outcome and follow-up

The MRI 6 months after diagnosis showed a complete resolution of the lesion (Fig. 1D). The patient continues to require thyroid, adrenal (hydrocortisone, 10 mg twice daily) and anti-diuretic hormone replacement therapy 6 months after her hospitalization. She continues to present with a secondary amenorrhea since her diagnosis, although at her most recent visit, her estrogen and gonadotropin levels suggested a recuperation of the gonadotropic axis. Her prolactin levels have returned to normal and the insulin-like growth factor 1 (IGF1) level remains slightly below the reference range. Additionally, she has a persistent but improved palsy of the sixth cranial nerve but no other neurological sequelae.

## Discussion

This case highlights a rare cause of secondary panhypopituitarism due to a pituitary lesion with a differential diagnosis of lymphocytic hypophysitis vs pituitary abscess in a context of decapitated meningitis. Both lymphocytic hypophysitis, with or without cystic components, and primary pituitary abscesses are exceedingly unusual conditions in the pediatric age group, but are potentially life threatening and require a prompt and complete hypothalamic pituitary evaluation.

Only ∼200 cases of primary pituitary abscesses have been reported in the literature, primarily in adults (2). Diagnosis may be difficult, as symptoms can be non-specific, without signs of an infectious process. Very few cases are diagnosed pre-operatively (3), although some cases have resolved without aspiration biopsy. Clinical symptoms are often non-specific and without signs of an infectious process. The most common symptoms are neurological (headache, visual impairment and cranial nerve palsy), but manifestations of hypopituitarism are frequent (central diabetes insipidus in 40%, hyperprolactinemia in 10% and other hormonal deficiencies in 20–30% of cases) (1) (3). Cerebral MRI showing a ring-enhancing cystic pituitary lesion, surgery and histopathology is helpful in the diagnosis, as well as microbiological cultures, with Gram-positive organisms (*Staphylococcus* spp. and *Streptococcus* spp.) being the most common pathogens. *Pseudomonas* spp. and *Klebsiella* spp., along with fungal and mycobacterial infections, have also been reported. Interestingly, cultures can be negative in 50–100% of cases (1) (3).

Treatment usually consists of a transsphenoidal evacuation and up to 8 weeks of i.v. antibiotics. In the absence of an identifiable pathogen, association of large spectrum antibiotics, such as third-generation cephalosporins, vancomycin and metronidazole, can be advocated. Prognosis is variable (*n*=24 cases), with 10% mortality rate, 60% complete remission and 30% partial remission. Of those in partial remission, persistent visual impairment or hormone deficiencies are reported, as well as relapse in about half of these cases (1). However, a recent series of 29 cases showed 0% recurrence rate (3). In most of the reported cases, mortality was associated with post-surgical complications such as meningitis, cerebritis, infectious vascular injury and CSF fistula.

Lymphocytic hypophysitis is equally rare, with 95 pediatric cases reported until 2011 (4). This incidence is much less compared with the 379 biopsy-proven cases in adults as of 2005 (5). According to a recent review by Kalra *et al*. (4), diabetes insipidus appears to be the most common hormonal deficiency in children accounting for 85% of cases; growth hormone deficiency was present in 76%, gonadal dysfunction in 32%, hypothyroidism in 30%, adrenal insufficiency in 21% and panhypopituitarism in 11% of children. The diagnosis of lymphocytic hypophysitis is based on laboratory evaluation (hormone levels, inflammatory markers and anti-pituitary antibodies), MRI findings (pear-shaped pituitary and cystic components) and histopathology (5). Interestingly, Japanese series of cases in adults revealed elevated IgG4 levels in serum and on pituitary biopsy specimens (6). The routine measurement of antibodies against anterior pituitary cell types or against arginine vasopressin-secreting cells is not recommended, as the former lack specificity and sensitivity and the latter are performed only within a research context (7). Glucocorticoids are the first-line treatment and are usually successful. Transsphenoidal surgery, more aggressive medication or radiosurgery has been used in more complicated cases (8). Mortality rates are reportedly low (8%) and presumably attributed to irreversible adrenal insufficiency, but 73% of reported cases need long-term hormonal replacement and relapses are quite common (5).

Although a cystic appearance is not classically described in cases of lymphocytic hypophysitis, isolated reports indicate that this is not an unusual presentation in imaging studies. In a case report of lymphocytic hypophysitis with cystic components at the MRI (9), the patient received anti-tuberculosis treatment before transsphenoidal evacuation of the abscess. The subsequent diagnosis of lymphocytic hypophysitis was based on immunohistochemical findings and the absence of detectable pathogens. No glucocorticoid treatment was initiated, as the surgery resulted in an immediate relief from the neurological signs. Combination of high-dose glucocorticoids and antibiotics is not a standard treatment for infectious pituitary abscesses, although prednisone (20 mg daily for 3 days) as part of routine preparation for transsphenoidal surgery has been used in adult patients without any adverse effects (2).

In our patient's case, neither the available immunohistochemical markers on pituitary biopsy nor the imaging findings helped to distinguish the two diagnoses, as they could be equally present in inflammatory processes of infectious or autoimmune origin. The radiological aspect of the lesion is more in favor of an infectious origin, but the absence of granulocytes in the liquid of the transsphenoidal biopsy is puzzling. Indeed, in the absence of specific diagnostic tools, a false diagnosis of lymphocytic hypophysitis can be present in other sellar lesions, cystic or not. The differential response to either antibiotics or glucocorticoids could confirm the origin of the pituitary lesion. Because of the severity of the neurological impairment, we decided to apply both treatments, with complete resolution of the pituitary lesion on follow-up MRI scans. The final diagnosis remains unclear.

Based on these observations and the favorable evolution of our patient, we conclude that a short course of high-dose glucocorticoids combined with antibiotics and neurosurgery is not harmful in cases where there is no diagnostic certainty as to the origin of a cystic sellar mass. This approach may also be beneficial, as it will diminish inflammation of either infectious or autoimmune origin while ensuring that the most likely pathogens are being targeted. The chance of a complete hormonal recovery is unlikely but will require continued follow-up by the endocrinologist.

## Patient consent

We confirm that we have obtained written informed consent from the patient (and the patient's parents) for publication of the submitted article and accompanying images.

## Author contribution statement

Dr D Manousaki wrote the first draft of the manuscript and did the review of the case and the literature search. N Alos, P Ovetchkine and C Mercier reviewed the patient on the ward and the patient who attended/is attending their outpatient clinics. C Deal and J J De Bruycker contributed to the manuscript revision, discussions and literature review. Each author listed on the manuscript has seen and approved the submission of this version of the manuscript and takes full responsibility for the manuscript.
